# Endothelial microRNAs in INOCA patients with diabetes mellitus

**DOI:** 10.1186/s12933-024-02331-x

**Published:** 2024-07-22

**Authors:** Marco Ferrone, Michele Ciccarelli, Fahimeh Varzideh, Urna Kansakar, Germano Guerra, Federica Andrea Cerasuolo, Antonietta Buonaiuto, Antonella Fiordelisi, Enzo Venga, Mafalda Esposito, Antonio Rainone, Roberto Ricciardi, Carmine Del Giudice, Fabio Minicucci, Tullio Tesorio, Valeria Visco, Guido Iaccarino, Jessica Gambardella, Gaetano Santulli, Pasquale Mone

**Affiliations:** 1 Casa di Cura “Montevergine”, Mercogliano, Avellino Italy; 2 University of Salerno, Baronissi, Italy; 3 Department of Medicine, Division of Cardiology, Albert Einstein College of Medicine, New York, USA; 4 Department of Medicine and Health Sciences “Vincenzo Tiberio”, University of Molise, Campobasso, Italy; 5 University of Naples “Federico II”, Naples, Italy; 6 Responsible Hospital, Campobasso, Italy; 7 ASL Naples, Napoli, Italy

## Abstract

Ischemia with non-obstructive coronary artery (INOCA) is a common cause of hospital admissions, leading to negative outcomes and reduced quality of life. Central to its pathophysiology is endothelial dysfunction, which contributes to myocardial ischemia despite the absence of significant coronary artery blockage. Addressing endothelial dysfunction is essential in managing INOCA to alleviate symptoms and prevent cardiovascular events. Recent studies have identified diabetes mellitus (DM) as a significant factor exacerbating INOCA complications by promoting endothelial impairment and coronary microvascular dysfunction. MicroRNAs (miRNAs) have emerged as potential biomarkers and therapeutic targets in various biological processes, including endothelial dysfunction and cardiovascular diseases. However, research on miRNA biomarkers in INOCA patients is sparse. In this study, we examined a panel of circulating miRNAs involved in the regulation of endothelial function in INOCA patients with and without DM. We analyzed miRNA expression using RT-qPCR in a cohort of consecutive INOCA patients undergoing percutaneous coronary intervention. We detected a significant dysregulation of miR-363-5p and miR-92a-3p in INOCA patients with DM compared to those without DM, indicating their role as biomarkers for predicting and monitoring endothelial dysfunction in INOCA patients with DM.

## Background

Ischemia with no-obstructive coronary artery (INOCA) is a leading cause of hospitalizations, driving adverse outcomes and reducing quality of life [1–6]. Endothelial dysfunction plays a crucial role in INOCA; indeed, despite the absence of significant coronary artery obstruction, in this pathologic condition endothelial cells fail to function optimally [7–10]. Such dysfunction contributes to the mismatch between myocardial oxygen supply and demand, leading to ischemia and symptoms like chest pain or discomfort. In particular, coronary microvascular dysfunction might be playing a crucial role in the onset of adverse events in INOCA subjects [11–14]. Understanding and addressing endothelial dysfunction is essential in managing patients with INOCA to improve symptoms and prevent adverse cardiovascular events [3, 4]. To counteract INOCA complications, our group has recently demonstrated that hyperglycemia is one of the mechanisms involved in its pathophysiology [15]. Diabetes mellitus (DM) and hyperglycemia are known to drive endothelial impairment and coronary microvascular dysfunction [16–18].

MicroRNAs (miRNAs, miRs) are short non-coding RNAs that post-transcriptionally regulate gene expression by binding to the 3′ untranslated region of target messenger RNAs (mRNAs), leading to its degradation or translational repression [19, 20]. It is currently accepted that miRs exert their activity in many biological processes and, as such, have been proposed as biomarkers and potential targets of novel therapeutic strategies [21, 22]; Moreover, miRs have been linked to endothelial dysfunction and cardiovascular diseases [23–27] and may be also useful to monitor the evolution of cardiovascular diseases and atherosclerosis [28–30].

However, currently there are no established biomarkers of endothelial dysfunction in INOCA patients. Hence, in our study, we monitored the expression of a panel of circulating miRs involved in the regulation of endothelial function in a population of individuals with a confirmed diagnosis of INOCA comparing patients with or without DM.

## Methods

We evaluated consecutive INOCA patients referred to the Casa di Cura “Montevergine”, Mercogliano (Avellino) and ASL Naples, both in Italy, for percutaneous coronary intervention (PCI). We defined DM according to the American Diabetes Association (ADA) guidelines [31]. Consistent with previous investigations [3, 4, 32], we defined INOCA as:

- Symptoms of myocardial ischemia;
- Non-obstructive coronary artery stenosis defined as: <50% diameter reduction and/or fractional flow reserve > 0.80;
- Objective evidence of myocardial ischemia;
- Impaired coronary microvascular function defined as: impaired coronary flow reserve (≤ 2.0), abnormal coronary microvascular resistance indices, coronary microvascular spasm, endothelial dysfunction with ≥ 20% luminal constriction during acetylcholine infusion, and/or coronary slow flow phenomenon.

The study was designed and conducted according to the principles outlined in the Declaration of Helsinki.

Circulating miRs were isolated from plasma samples, obtained using EDTA-containing tubes. We extracted miRs using the miRNeasy Serum/Plasma kit (*Qiagen*, Hilden, Germany) according to the protocol provided by the manufacturer. RNA purity and concentration were evaluated by spectrophotometry using a NanoDrop ND-2000 (*ThermoFisher*, Waltham, MA); reverse transcription was performed using the miRCURY LNA Universal RT miR PCR kit (Qiagen); miRs expression was analyzed by RT-qPCR, as we described [27]. The quality of miRs was determined using the Agilent Small RNA Kit [33]. We analyzed a panel of miRs that we have previously demonstrated to be involved in the regulation of endothelial dysfunction [26]; miR expression was analyzed by quantitative real-time polymerase chain reaction (RT-qPCR), as described [26]. The RNA Spike-in kit (*Qiagen*) was used as an exogenous control of RNA extraction following the manufacturer’s instructions. For quality control, we used three synthetic RNA spike-ins (UniSp2, UniSp4, and UniSp5) at different concentrations; as a control for cDNA synthesis, we used UniSp6 spike-in and cel-miR-39-3p.

Relative gene expression was determined using the 2-ΔΔCT method [26]. To normalize, we first selected the miRs that displayed the least variability in their cycle threshold (Ct) values in all samples using the *geNorm* and *NormFinder* bioinformatic algorithms [34, 35], which revealed that the most stable (less variable when comparing the two groups) miRs were miR-125a-5p and miR-19a-5p. Then, we used the *BestKeeper* method [36] to calculate the geometric mean of the pair-wise Ct values (Ct values of miR-125a-5p and miR-19-5p); the relative expression data were calculated with the Ct of each target miR with reference to the *BestKeeper* value. To confirm the absence of hemolysis in our samples, we assessed the presence of cell free hemoglobin at the spectrophotometer measuring absorbance at 414 nm (A414). Since dyslipidemia could interfere with this kind of evaluation, we also measured the delta quantification cycle (ΔCq) of known blood cell-associated miRs (miR-16-5p and miR-451a) and a control miR (miR-23a-3p) that is known to be invariant in plasma affected by hemolysis.

### Statistical analysis

All data were analyzed using the GraphPad Prism software v. 9.0 (*Dotmatics*, Boston, MA, USA). Data are expressed as means ± SD or as numbers and percentages. The differences in miR levels were analyzed using two-tailed t-tests as appropriate after having verified the normal distribution of values via Kolmogorov–Smirnov test.

## Results

**Fig. 1:**
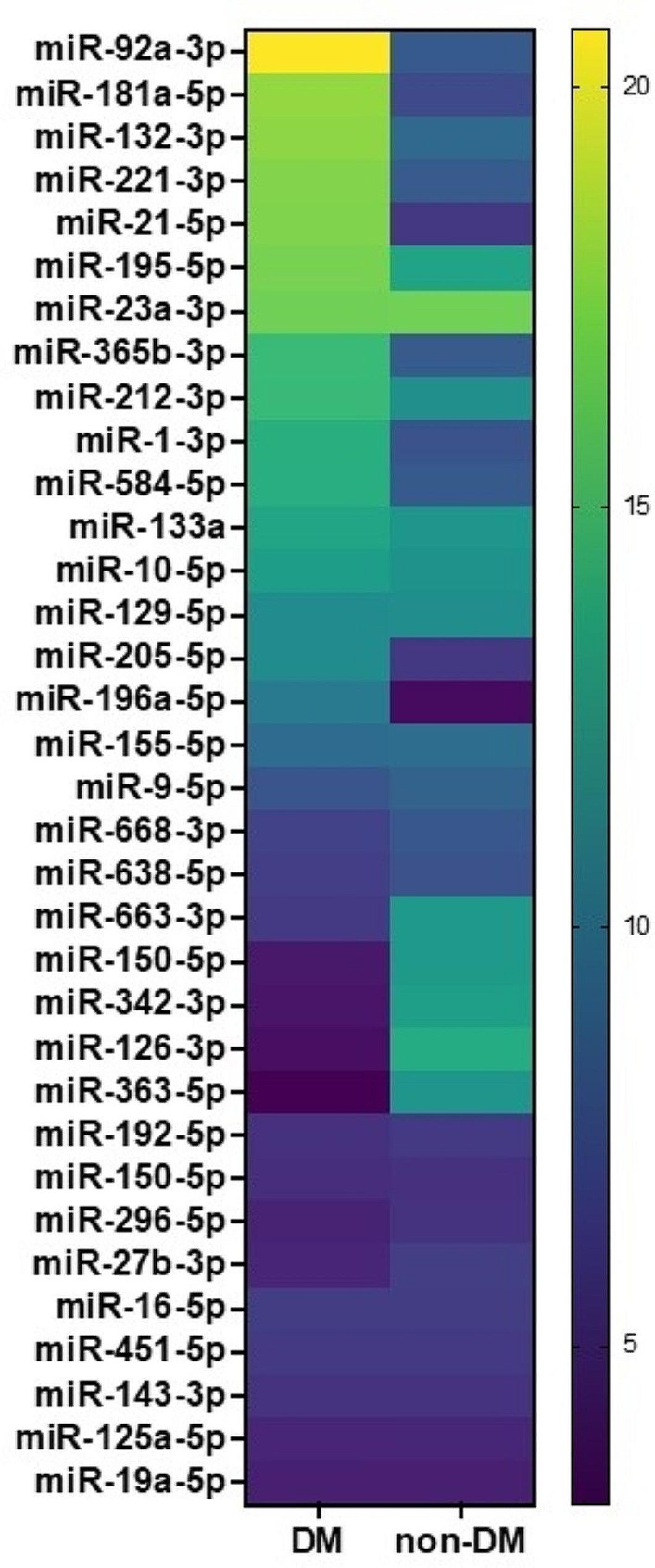
Heat-map of the expression of circulating miRNAs in the indicated groups of patients

42 patients agreed to enter our study (28 without DM, 14 with DM). Clinical characteristics are shown in Table 1. We measured the expression levels of the panel of miRs that we had previously validated [26] and in INOCA patients with DM (compared to patients without DM) we detected an increased expression of several miRs that have been previously associated with endothelial dysfunction, and a reduced expression of miRs that have been shown to be protective in terms of endothelial function (see heat map in Fig. 1).

**Table 1:** Clinical characteristics of our INOCA patients

	No-diabetes	Diabetes
N	28	14
Mean age (years)	68.3 ± 10.5	73.1 ± 11.7
Fasting plasma glucose (mg/dL)	99.6 ± 12.7	179.1 ± 41.2*
HbA1c (%)	5.8 ± 0.4	7.3 ± 0.8*
Creatinine (mg/dL)	1.0 ± 0.2	1.0 ± 0.3
Total Cholesterol (mg/dL)	179.2 ± 27.4	182.5 (30.3)
Hypertension	24 (85.7)	11 (78.6)
Dyslipidemia	20 (71.4)	9 (64.3)
Smoking	11 (39.3)	2 (14.3)
Atrial fibrillation	4 (14.3)	2 (14.3)
COPD	5 (18.0)	3 (22.0)

Data are means ± SD

* COPD* chronic obstructive pulmonary disease

**Fig. 2:**
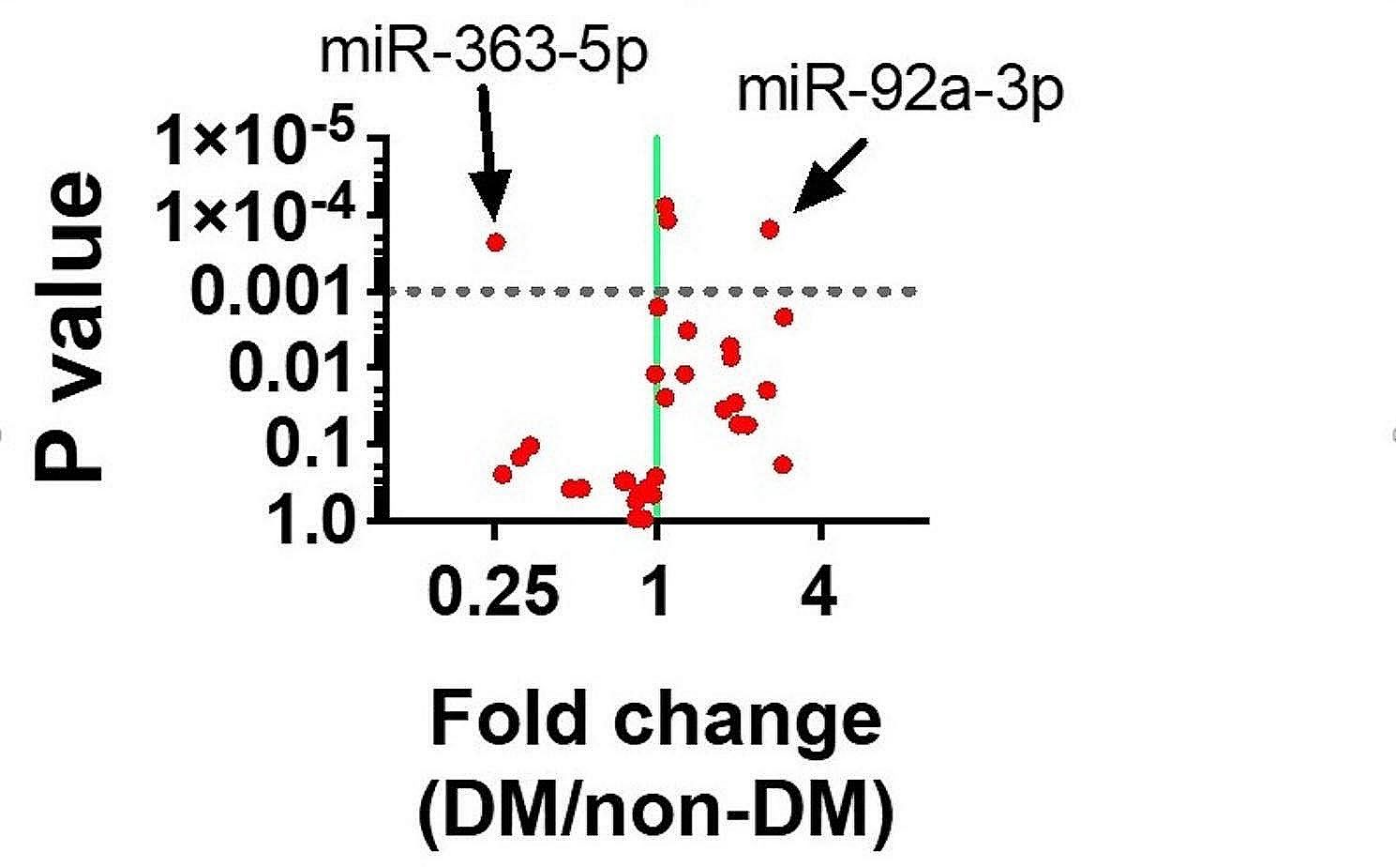
Volcano plot depicting miRNA analyses comparing DM vs. non-DM INOCA patients. The horizontal dotted line represents a P value of 0.001; thus, the points in the plot above that line represent the differentially expressed miRNAs with statistical significance

Then, we analyzed which of these miRs was significantly upregulated or downregulated when comparing INOCA patients with DM to INOCA patients without DM. We found that miR-363-5p was significantly downregulated (*P* < 0.001), whereas miR-92 was significantly upregulated (*P* < 0.001), in DM subjects, as shown in the volcano plot depicted in Fig. 2.

## Discussion

In the present study, we have identified, for the first time to our knowledge, circulating miRs involved in endothelial dysfunction that could be useful for monitoring INOCA patients with DM.

Endothelial dysfunction is a hallmark of diabetic vascular complications. In DM, dysregulated miR expression contributes to endothelial dysfunction through various mechanisms, including the modulation of pathways involved in endothelial fitness, inflammation, oxidative stress, and angiogenesis. For example, miR-126, miR-155, and miR-21 have been implicated in regulating endothelial cell function and angiogenesis by targeting genes involved in endothelial nitric oxide synthase (eNOS) signaling, inflammatory pathways, and vascular endothelial growth factor (VEGF) signaling [37].

Furthermore, miRs can influence endothelial integrity by targeting genes involved in oxidative stress and inflammation [38, 39], both of which are key drivers of endothelial dysfunction in DM. Emerging evidence suggests that circulating miRs may serve as biomarkers for endothelial dysfunction and diabetic vascular complications; in fact, several studies have identified dysregulated miR expression profiles in the circulation of diabetic patients with endothelial dysfunction [40–42], thereby providing insights into the pathogenesis and progression of diabetic vascular complications.

The identification of two miRs, namely miR-92 and miR-363-5p, that are differently expressed in INOCA patients with and without DM, is fully consistent with previous reports. Endothelial-derived extracellular miR-92a promotes arterial stiffness by regulating phenotype changes of vascular smooth muscle cells and reduces oxidative stress [43, 44]. On the other hand, miR-363-5p reduction results in a significant decrease in endothelial cell tube formation [45]. Henceforth, the aforementioned miRs may be useful markers in the management of INOCA patients, with or without diabetes. Emphasizing the novelty of our work, we did not find any other study investigating miRs in INOCA patients.

The relationship between DM, endothelial dysfunction, and the pathogenesis of INOCA is complex and multifaceted. Indeed, endothelial dysfunction, characterized by impaired endothelium-dependent vasodilation and pro-inflammatory and pro-thrombotic states, is a common feature of both DM and INOCA [46, 47]. In DM, chronic hyperglycemia, insulin resistance, and dyslipidemia further contribute to endothelial dysfunction through various mechanisms, including increased oxidative stress, inflammation, and activation of the renin-angiotensin-aldosterone system [48, 49]. Of note, coronary slow flow (CSF), assessed by invasive coronary angiography has been frequently seen as a potential indicator of coronary microvascular dysfunction in INOCA patients [50–52]; however, a recent prospective study has demonstrated that CSF is not a reliable angiographic surrogate of abnormal coronary flow reserve (CFR) or index of microcirculatory resistance (IMR) [53].

Endothelial dysfunction and DM play central roles in the pathophysiology of INOCA by promoting coronary microvascular dysfunction, characterized by impaired vasodilation, increased vasoconstriction, and altered microvascular structure [46, 54, 55]. Dysfunction of the coronary microvasculature results in inadequate myocardial perfusion despite the absence of significant obstructive coronary artery disease, leading to angina-like symptoms and ischemia [56, 57]. Additionally, impaired endothelial function in DM is associated with enhanced endothelial permeability and increased vascular inflammation, which may contribute to myocardial injury and fibrosis, further compromising myocardial perfusion and function in INOCA [58, 59]. DM-related endothelial dysfunction may also exacerbate, and interact with, other risk factors for INOCA, such as hypertension, obesity, and dyslipidemia [60], further aggravating coronary microvascular dysfunction and ischemia. Again, we are the first group to highlight the importance of miR profiles in INOCA.

Nevertheless, our work is not exempt from limitations, including the small size of our population and having limited the panel of miRs to the ones that were implied in endothelial dysfunction. Equally important, further research is needed to elucidate the specific mechanisms underlying the exact role of the miRs that we have demonstrated to be differently expressed in DM and non-DM INOCA patients.

## Conclusions

Understanding the role of miRs in endothelial dysfunction may provide novel insights into the pathogenesis of diabetic vascular complications and offer potential therapeutic targets for intervention in INOCA and other diabetic vascular complications.

## Data Availability

The data that support the findings of this study are available from the last author upon reasonable request.
